# Post-traumatic endophthalmitis caused by *Nocardia nova*

**DOI:** 10.1099/jmmcr.0.005175

**Published:** 2019-02-20

**Authors:** Jesús Rodríguez-Lozano, Carlos Armiñanzas Castillo, Carlos Ruiz de Alegría Puig, Juan Antonio Ventosa Ayarza, Maria Carmen Fariñas, Jesús Agüero, Jorge Calvo

**Affiliations:** ^1^​ Departament of Microbiology, University Hospital Marqués de Valdecilla - IDIVAL, Santander, Spain; ^2^​ Department of Infectious Diseases, University Hospital Marqués de Valdecilla, Santander, Spain; ^3^​ Department of Ophthalmology, University Hospital Marqués de Valdecilla, Santander, Spain; ^4^​ Department of Medicine and Psychiatry, University of Cantabria, Santander, Spain; ^5^​ Department of Molecular Biology, University of Cantabria, Santander, Spain

**Keywords:** *Nocardia*, *Nocardia nova sensu stricto*, *Nocardia nova* complex, endophthalmitis, ocular nocardiosis

## Abstract

**Introduction:**

*Nocardia nova* complex has been associated with infections in both immunocompetent and immunocompromised patients. Infection can be localized or disseminated, affecting skin and soft tissues, the respiratory system, bones and joints, the circulatory system and especially the central nervous system. Ocular infections such as keratitis, scleritis, conjunctivitis, dacryocystitis, orbital cellulitis and endophthalmitis due to *Nocardia* spp. are infrequently reported, and usually described after penetrating corneal trauma or ocular contact with plants and soils.

**Case presentation:**

An immunocompetent male presented with a history of penetrating ocular trauma that had evolved to infectious endophthalmitis, which was refractory to different antibiotic treatments. No micro-organisms were isolated from repeated conjunctival smear and corneal scraping cultures between the ocular trauma (August 2014) and the endophthalmitis diagnosis (November 2015). After this period, *N. nova sensu stricto* was isolated in aqueous humour aspirate. Treatment was adjusted and clinical improvement was obtained after an adequate microbiological procedure, including an optimal sampling and an antimicrobial-susceptibility testing report.

**Conclusion:**

*Nocardia* identification to the species level and performance of antimicrobial-susceptibility tests are both essential tools for treatment adjustment and clinical improvement.

## Introduction

*Nocardia* species can cause infections in immunocompetent and immunocompromised patients, usually by inhalation from environmental sources, and lung disease is the most reported clinical form [1]. We present a clinical case of endophthalmitis by *Nocardia* nova *sensu stricto* in an immunocompetent male with a history of penetrating ocular trauma more than a year before. We argue that it is possible that the *Nocardia* micro-organism was present in superficial ocular lesions, before the endophthalmitis manifested a year later, and so its presence was not detected in the microbiological culture of the superficial samples (ocular smears and corneal scrapings) due to the short period of incubation, the presence of mixed flora, the previous use of antibiotics or an inadequate sample collection. By presenting this case, we want to reinforce the importance of a good communication between clinical practitioners and microbiological staff to obtain an adequate sample and the most precise diagnosis.

Moreover, as we confirm with this clinical picture, treatment of *Nocardia* infections should be individualized, so correct identification and antimicrobial-susceptibility testing are critical [1]. Recent incorporation of more advanced identification methods offers an opportunity for rapid and correct identification at the species level [2], which favours a more adequate therapy.

## Case Report

A 66-year-old man presented to the hospital emergency department in August 2014 complaining of redness and pain in his left eye, with blurred vision. His past medical history revealed no human immunodeficiency virus infection or other immunodeficiency disorders. The patient reported a history of perforating trauma to his left eye with a splinter of a plastic hose while working in the countryside. On examination, a central corneal wound was detected, which was self-sealing but opened up to pressure. A positive Tyndall effect was also observed. Topical (0.5 % moxifloxacin 1 eye drop/5 h) and oral (ciprofloxacin, 500 mg/8 h)) antibiotics were prescribed for the following 6 months. Intravenous (IV) vancomycin (1 g/12 h) and ceftazidime (1 g/8 h) treatment was administered for 6 weeks. Samples of corneal scrapings and conjunctival smears sent to the microbiology department yielded no relevant results, and an anatomopathological study of the cornea did not present conclusive data. Given the torpid evolution, a cornea transplant was finally performed in June 2015.

**Fig. 1.**
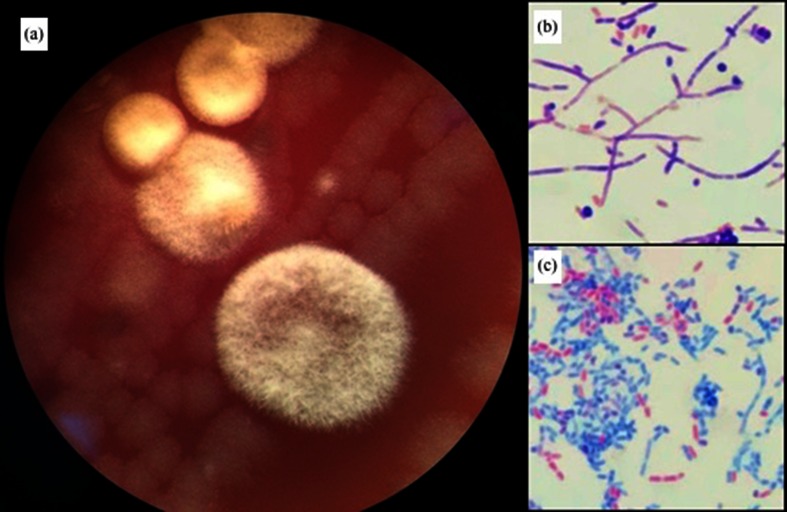
Strain morphology after 72 h incubation. (a) Blood agar (viewed with a magnifying glass, ×40 magnification). (b) Gram stain (x100 magnification). (c) Ziehl–Neelsen stain (x100 magnification).

The patient was admitted again to the hospital on November 2015 due to an endophthalmitis in his left eye, despite having been treated with 0.5 % moxifloxacin and 0.1 % dexamethasone drops since the cornea transplant. IV vancomycin (1 g/12 h) and ceftazidime (1 g/8 h) were prescribed, in addition to topical (1% drop/2 h) and oral voriconazole (200 mg/12 h). An aspiration from the anterior chamber of the eye was performed, and an aqueous humour sample was sent to the microbiology department. Bacterial culture was performed on Columbia blood agar, chocolate agar, McConkey agar and in thioglycolate broth with incubation at 35±2 °C in atmospheric conditions supplemented with 5 % CO_2_. After 72 h, slightly whitish dry-looking colonies were observed on blood agar and on chocolate agar (Fig. 1). Gram staining yielded branched Gram-positive rods, and modified Ziehl–Neelsen stain revealed acid-fastness. The micro-organism was initially identified as *Nocardia asteroides* (99.9 % identity) using matrix-assisted laser desorption/ionization-time of flight (MALDI-TOF) MS (Vitek MS; bioMèrieux). Sequencing of the 16S rRNA gene and analysis using blast (http://www.ncbi.nlm.nih.gov) showed 99.9 % similarity to *N. nova* ATCC 33726 (GenBank accession number: NR115835.1). Subsequently, applying an updated software version for the MALDI-TOF MS, the isolate was identified as *Nocardia africana/N. nova*. Antimicrobial-susceptibility testing was performed using Etest strips (bioMèrieux) [3] and susceptibility clinical categories were defined according to Clinical and Laboratory Standards Institute (CLSI) guidelines for mycobacteria, nocardiae and other actinomycetes [4]. The micro-organism was reported as susceptible to ceftriaxone (MIC=0.5 mg l^−1^), imipenem (0.02 mg l^−1^), amikacin (1 mg l^−1^), clarithromycin (0.03 mg l^−1^) and linezolid (0.016 mg l^−1^), and resistant to benzyl-penicillin (>32 mg l^−1^), amoxicillin-clavulanic acid (>256 mg l^−1^), tobramycin (128 mg l^−1^), ciprofloxacin (>32 mg l^−1^), moxifloxacin (8 mg l^−1^) and co-trimoxazole (>32 mg l^−1^). Although no susceptibility breakpoints have been established for vancomycin by CLSI, the MIC value was high (8 mg l^−1^).

After the microbiological diagnosis of endophthalmitis caused by *N. nova*, the patient was prescribed amikacin, 1 g/24 h/IV, and imipenem, 500 mg/6 h/IV, for 2 months and discharged. Treatment was continued with ambulatory amikacin, 1 g/24 h/IV, and ceftriaxone, 2 g/24 h/IV, for 1 month; linezolid, 600 mg/12 h/orally for another month; and clarithromycin, 500 mg/12 h/orally for 2 more months (total treatment time: 6 months). The patient was followed-up with consultations with the ophthalmology and infectious diseases departments for 2 years, and satisfactory evolution was observed.

## Discussion

*N. nova* complex (including the species: *N. nova sensu stricto*, *N. africana*, *Nocardia aobensis*, *Nocardia elegans*, *Nocardia kruczakiae* and *Nocardia veterana*) has been associated with infections in both immunocompetent and immunocompromised patients. In a recent review, it was reported as the second most prevalent *Nocardia* species recovered in clinical samples from Spain [5]. It can affect the skin and soft tissues [6], respiratory system [7], bones and joints [8], circulatory system [9], and central nervous system [10]. Ocular infections such as keratitis, scleritis, conjunctivitis, dacryocystitis, orbital cellulitis and endophthalmitis due to *Nocardia* spp. are infrequently reported [11–13] and usually described after penetrating corneal trauma or ocular contact with plants and soils. They are characterized by loss of vision, eye discomfort or pain, and small whitish lesions in the case of keratitis [12].

Although is not possible to elucidate whether the endophthalmitis in this case had surgical (5 months before) or traumatic (1 year before) origin, we consider the last option as the most probable due to the persistent symptoms since the beginning. The use of different antimicrobial agents (moxifloxacin, ciprofloxacin, gentamicin, vancomycin and voriconazole), to which the micro-organism is reported to be resistant, before the definitive microbiological diagnosis could support this assertion in the clinical picture. The infection was resolved only when an adequate antimicrobial therapy was administered more than 1 year later. Is it possible that the *Nocardia* isolate was present in the first months? If so, why was the micro-organism not detected in conventional culture? A plausible reason is the type of sample sent to the laboratory, consisting of superficial samples such conjunctival smears and corneal scrapings. In the case of conjunctival smears, the incubation time approved in our procedures for the conventional culture of conjunctival samples is only 48 h, as long as no relevant comments are reflected on the microbiological request. Corneal scraping is a good sample, but its sensitivity is not always optimal, especially in patients with deep-seated keratitis, and depends on the amount of sample collected and the location. Moreover, usually the inoculum in corneal sample is so low that it is affected by the presence of topical antibiotics, reducing bacterial culture sensitivity significantly. However, when the infection evolved to a more severe and evident endophthalmitis, and an aqueous humour sample was obtained, the causative agent could be diagnosed and adequate therapy initiated. Based on this case, we suggest a prolonged incubation time for samples related to ocular trauma infections and contact with the microbiologist in case of negative cultures/results.

Although microbiological identification of the genus *Nocardia* is usually easy to perform, accurate species identification following standard protocols is difficult. In our case, definitive identification was performed by sequencing the 16S rRNA gene. However, in order to evaluate MALDI-TOF MS as a new diagnostic tool, two different MALDI-TOF MS software versions were used. At the time of micro-organism identification in the clinical case, the MALDI-TOF MS software version (Vitek MS v2) only included *N. asteroides* identification within the genus *Nocardia*. The current software version recently available in our Microbiology laboratory (Vitek MS v3) includes *Nocardia abscessus*, *N. africana/N. nova*, *Nocardia beijingensis*, *Nocardia brasiliensis*, *Nocardia cyriacigeorgica*, *Nocardia farcinica*, *Nocardia neocaledoniensis*, *Nocardia otitidiscaviarum*, *Nocardia paucivorans*, *Nocardia pseudobrasiliensis*, *Nocardia transvalensis* and *N. veterana*. Applying this latest software, the strain (previously stored by freezing) was identified as *N. nova/*N. *africana*. Continuous updating of databases supports and reinforces MALDI-TOF MS technology as a promising tool for identifying actinomycete micro-organisms.

**Fig. 2.**
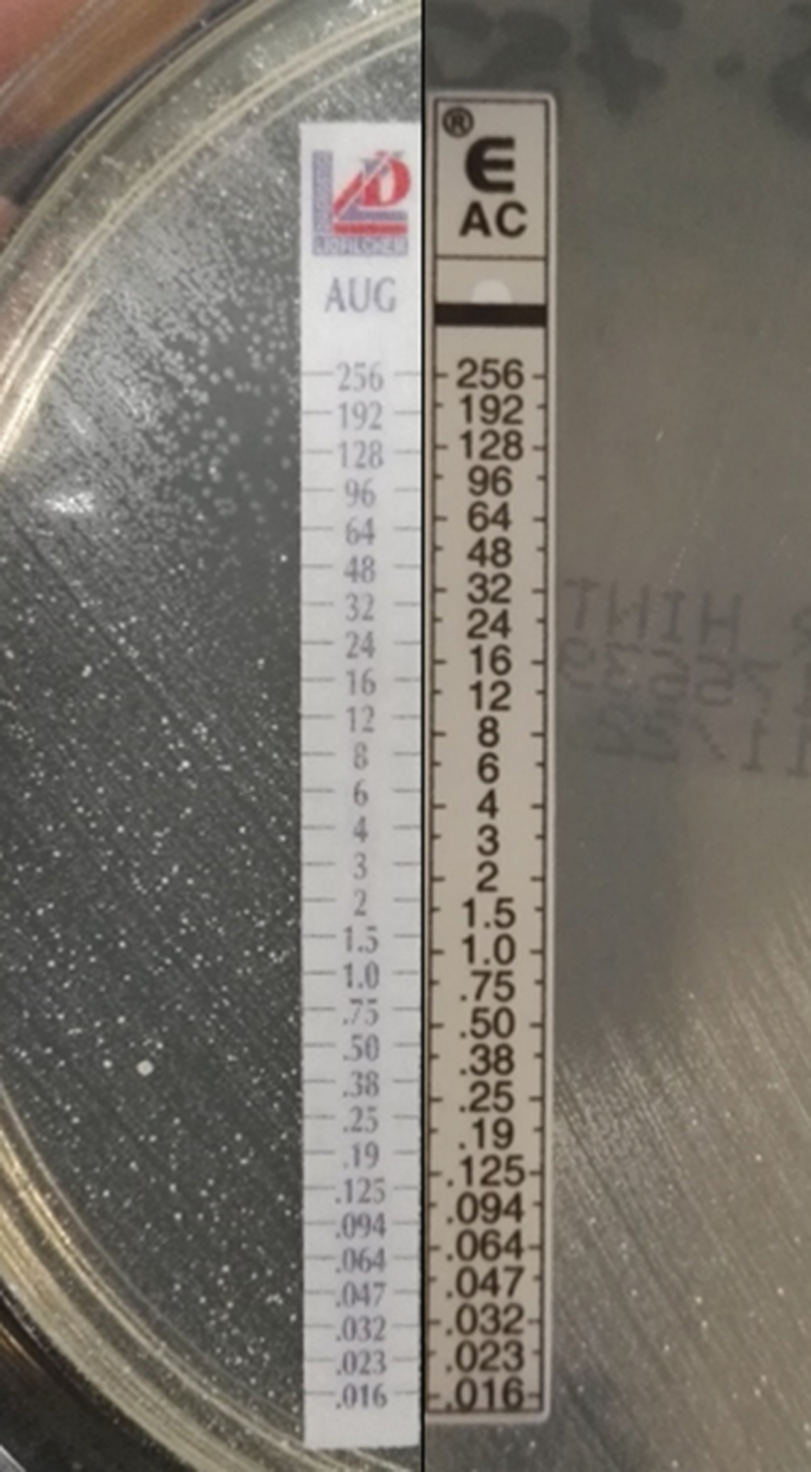
Susceptibility to amoxicillin (AC) and resistance to amoxicillin-clavulanic acid (AUG) (inducible β-lactamase).

*N. nova* complex presents the susceptibility pattern described for the *N. asteroides* pharmacological group III, characterized by susceptibility to amoxicillin but resistance to amoxicillin-clavulanic acid (β-lactamase inducible by clavulanic acid), and susceptibility to erythromycin, amikacin, ceftriaxone and linezolid [14]. Fluoroquinolone resistance is common. This characteristic susceptibility pattern matched completely with our micro-organism (Fig. 2).

The treatment of infection by *N. nova* often requires the combination of systemic antibiotic therapy and surgical management [6, 8]. IV antibiotic agents should be initially administered for 6–8 weeks, preferably with two antimicrobials with confirmed *in vitro* susceptibility, including co-trimoxazole [7]. Because of possible resistance to this last compound [14], as in the case we report, the combination of co-trimoxazole, amikacin and ceftriaxone or imipenem has been recommended [8, 12] for empirical treatment. Once co-trimoxazole susceptibility is confirmed by a laboratory, it is possible to continue with oral co-trimoxazole monotherapy for at least 3–6 months, depending on the focus of infection and the immune status of the patient. Oral alternatives, such as macrolides and linezolid [5], have also been reported.

It is a recognized fact that infections caused by these micro-organisms pose diagnostic and therapeutic challenges. A high degree of clinical suspicion will help early diagnosis by microbiologists and the administration of appropriate therapy.
